# Evaluation of in-service speed performance improvement by means of FDR-AF (frictional drag reducing anti-fouling) marine coating based on ISO19030 standard

**DOI:** 10.1038/s41598-020-80107-5

**Published:** 2021-01-13

**Authors:** Yurim Cho, Kyung Hwan Jeon, Sang Bong Lee, Hyun Park, Inwon Lee

**Affiliations:** 1 Department of Naval Architecture and Ocean Engineering, Pusan National University, Busan, 46241 Korea; 2 POS SM Co., Ltd., Busan, 48938 Korea; 3 Lab021 Co. Ltd., Busan, 48508 Korea

**Keywords:** Chemistry, Engineering

## Abstract

In previous reports by the authors, the drag reduction performance of a novel frictional drag reduction self-polishing copolymer (FDR-SPC) was presented. The drag-reducing functional compound polyethylene glycol methacrylate (PEGMA) was used in the synthesis process, thereby allowing the release of polyethylene glycol (PEG) into seawater by a hydrolysis reaction. In a laboratory skin friction measurement, a low-friction antifouling (AF) coating based on FDR-SPC was found to provide a 25% skin friction reduction compared with a conventional AF coating. This coating was then applied to the entire underwater surface of a 176 k DWT bulk carrier during dry docking in December 2015. The propulsion performance of the present vessel as well as the weather conditions were recorded over five years from November 2014 to December 2019. It was imperative that the hull coating performance be evaluated without being affected by the additional resistance component associated with weather conditions such as wind and waves. ISO 19,030 is proposed as a new international standard for that purpose. Based on this standard, in-service navigation data collected from the 176 k DWT bulk carrier, which amounts to 5.7 million data points, are analyzed to assess the speed improvement performance of the present frictional drag reduction antifouling (FDR-AF) coating. The analysis results indicate that the present coating leads to a speed increase of 3.72% over the conventional AF coating. The speed improvement effect is equivalent to power (fuel) saving of 11.7%.

## Introduction

The reduction in frictional drag of ships is of prime importance in terms of ship energy economy. The greatest energy demand on ships is to overcome drag during navigation, which consists of frictional, pressure and wavemaking components. Owing to the improvement of modern hull form design techniques, the wave and pressure drag components can be maintained at less than 20 percent of the total resistance in most ship types^1^. Consequently, the impact of frictional drag reduction (FDR) of ships becomes more conspicuous. For instance, Park and Lee^1^ pointed out that a 10% reduction in frictional drag would lead to savings of 4.7 billion US$/year, based on international marine bunker statistics^2^.

The prevalence of skin frictional drag of ship hulls is due to energetic turbulent momentum transfer, which is caused by coherent structures of the boundary layer flow, e.g., hairpin vortices. Various control strategies for the attenuation of such coherent structures have been proposed over several decades. Despite the potential advantage and diversity of control strategies, however, the implementation of such methods on marine vessels has essentially been restricted to a limited range. Air injection, also known as air lubrication, is the most notable example of turbulent skin FDR application, yielding approximately 10% net energy savings^3,4^. Note that the air injection method is an active method in which the air blowing apparatus needs to be installed on the ship. This capital investment could be regarded as a drawback in terms of cost benefit analysis.

Another effective drag reduction strategy is polymer injection, which was first introduced by Toms^5^, who found that the addition of a few ppm of a high-molecular-weight polymer to a turbulent water flow can result in a large (up to 80%) reduction in skin friction drag. Long chain polymer molecules dissolved in water extract turbulent energy from the adjacent flow by coiling their chain structures and then release energy by stretching back in the shear flow. Various studies have investigated the drag reduction efficiency of polymer injection in turbulent boundary layers^6–8^. From the aspect of implementation, however, polymer injection is impractical for ship applications. The cost benefit ratio involved with the costly polymer solution is worse than that for air injection. In addition, diffusive mass transfer of polymer out of the wall could degrade the FDR effect with increasing downstream distance from the injection hole. Inspired by the homogeneous releasing capability of antifouling (AF) coatings, Yang et al*.*^9^ proposed a polyethylene oxide (PEO)-containing AF coating as a feasible alternative to the injection method. It was found from various laboratory tests that this coating exhibited significant drag reduction efficiency in excess of 10%. In that coating, however, the PEO powders were physically mixed with the coating matrix, thereby giving rise to an increase in surface roughness associated with the rapid release of highly dissolvable PEO powder. This may be detrimental to the longevity of drag reduction performance.

**Figure 1:**
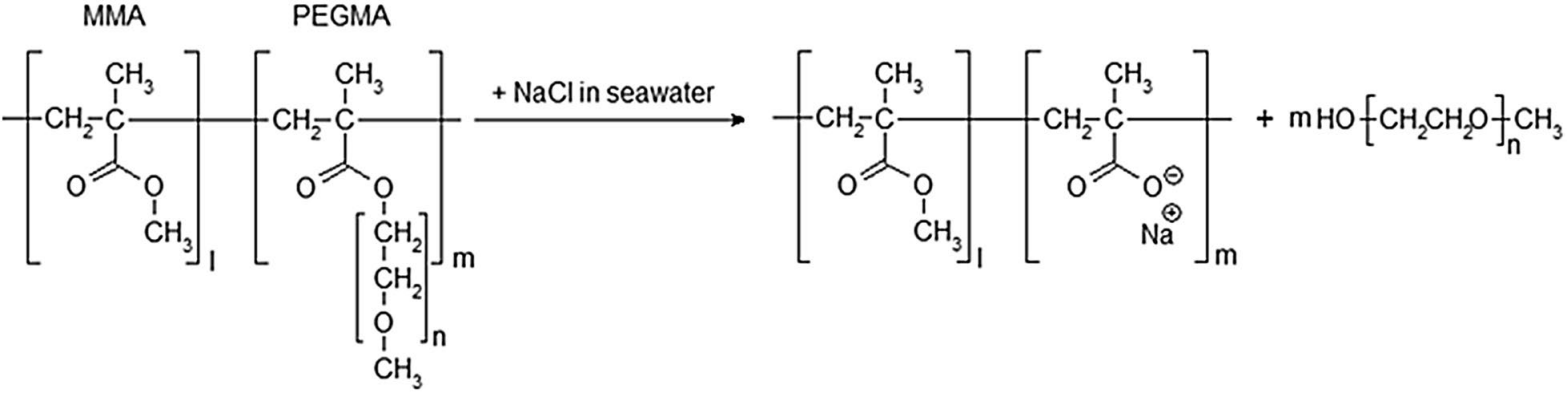
Hydrolysis reaction of FDR-SPC.

To overcome the drawback of the PEO-mixed coating in previous research, a novel frictional drag-reducing self-polishing copolymer (FDR-SPC) was first synthesized in a previous study by the authors^10,11^. Self-polishing copolymer (SPC), a polymer resin, is a major constituent of the coating film matrix and forms a gradually dissolvable layer through a hydrolysis reaction with water. Lee et al*.*^10^ devised FDR-SPC through a series of synthesis processes in which skin friction-reducing functional radicals, such as polyethylene glycol methacrylate (PEGMA), are copolymerized with other monomers, such as methyl methacrylate (MMA) and zinc methacrylate (ZMA). Since PEGMA is chemically bonded to the resin, its release rate can be controlled by the hydrolysis reaction of FDR-SPC, thereby ensuring prolonged skin friction reduction. Figure 1 depicts the release mechanism of PEO from the hydrolysis reaction between FDR-SPC and seawater.

Lee et al*.*^10^ reported that FDR-SPC exhibits skin friction reduction as large as 13.5% over smooth surfaces. In addition, a significant reduction in the turbulence intensity for FDR-SPC was demonstrated, corroborating the presence of the Toms effect caused by FDR-SPC. Furthermore, an FDR-AF coating, which is an AF coating manufactured from FDR-SPC, showed a stable FDR performance in the torque measurement test for a rotor operating for six months. This FDR-AF coating was subsequently commercialized by a local marine coating manufacturer. After the AF efficiency had been confirmed through the patch test for a couple of years, this product was chosen to be applied on the whole underwater surface of a 176 k DWT bulk carrier to evaluate the full-scale energy saving performance during service. The dry docking was finished in December 2015.

Since marine AF coating is indispensable for all ocean-going vessels and devoid of any moving part for injection, the present FDR-AF can be regarded as an ideal platform to implement the skin friction reduction technique in ships, provided that the full-scale energy saving performance can be verified during service. With the aim to confirm the energy saving performance of the present FDR-AF coating, a four-year-test campaign was carried out; during the first three-year period, the propulsive performance of the 176 k DWT bulk carrier with the present FDR-AF coating was monitored. After finishing the three-year service period in December 2018, the bulk carrier was then repainted with conventional AF coating, and subsequent operation was recorded for another year. The data in the fourth year with the conventional AF coating are utilized as a control period with which the first year data with the FDR-AF coating are compared under the same fresh-coated, clean hull surface condition. Thus, great care was undertaken in this study to isolate and quantify the coating effect on ship propulsive performance. The entire process of full-scale ship performance analysis in this study is based on the international standard ISO19030, which is described in the following section.

## Vessel information and ISO19030 standard

### Vessel information

The vessel under consideration is a 176 k DWT bulk carrier, of which principal particulars are listed in Table 1. Operational data have been collected automatically since the onboard ship performance management system (SPMS) was installed in November 2014.

**Table 1:** Principal particulars of the 176 k DWT bulk carrier.

Designation	Symbol	Value (m)
Length overall	LOA	291.80
Length between perpendiculars	LBP	282.20
Breadth	B	45.00
Depth	D	24.75
Mean draft, laden condition	T_L_	18.25
Mean draft, design condition	T_D_	16.50
Mean draft, ballast condition	T_B_	7.95

**Figure 2:**
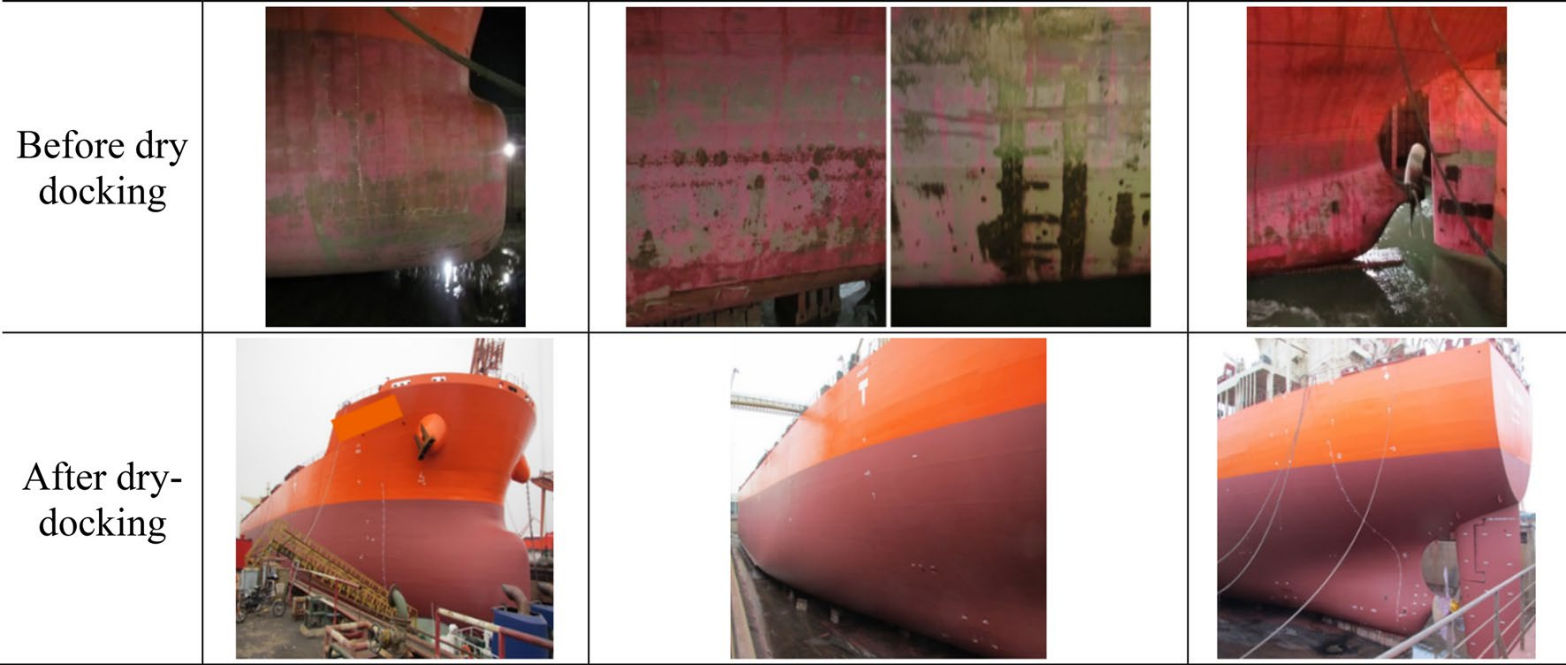
Photographs of the 1st dry docking (November 2015).

**Figure 3:**
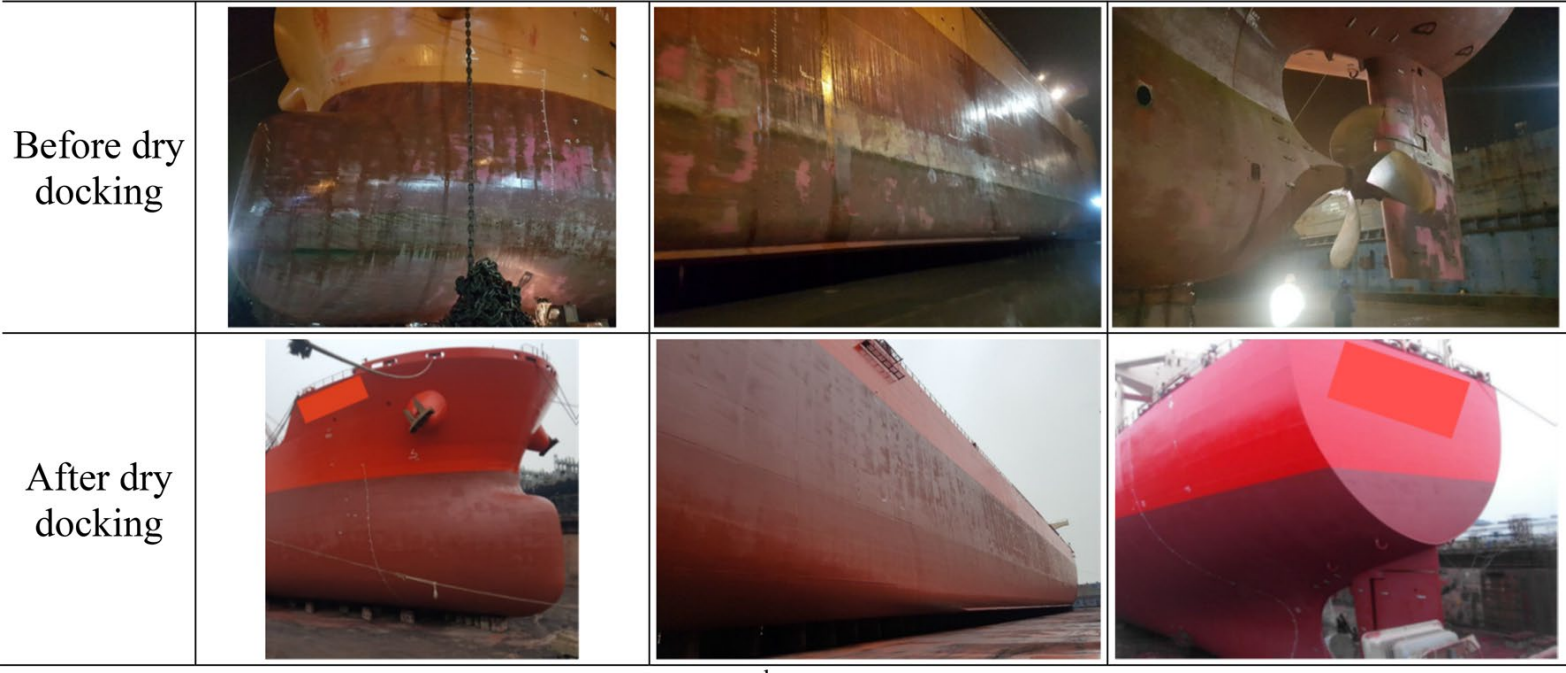
Photographs of the 2nd dry docking (December 2018).

Table 2 lists important events in chronological order. The present vessel underwent dry docking twice, in November 2015 and December 2018. At the 1^st^ dry docking as shown in Fig. 2, the conventional AF coating was removed, and then the FDR-AF coating was applied on the entire underwater hull area. In contrast to the heavily damaged and fouled surface conditions before dry docking, the hull surface after dry docking exhibited fresh and clean conditions. At the 2nd dry docking in Fig. 3, the previously applied FDR-AF was removed, and the hull was coated again with conventional AF coating. Again, the hull surface condition reverted to clean upon redocking. Consequently, it is foreseeable that the speed performance of the ship was considerably improved after dry docking, regardless of how the AF coating was changed.

**Table 2:** Key events and service periods for the 176 k DWT bulk carrier.

Event	Time/period	AF coating	Voyage no
SPMS installation	Nov. 2014		
1st service period (5 years, data available only for the 5th year after SPMS installation)	Dec. 2014 ~ Nov. 2015	Conventional	Ballast 29 ~ 37/laden 29 ~ 37
1st dry docking	Nov. 2015		
2nd service period (3 years)	Nov. 2015 ~ Nov. 2018	FDR	Ballast 38 ~ 63/laden 38 ~ 63
2nd dry docking	Dec. 2018		
3rd service period (3 years, data analyzed only for the 1st year)	Jan. 2019 ~ Dec. 2019	Conventional	Ballast 64 ~ 75/laden 64 ~ 75

Between the 1st and 2nd dry docking cycles, the 2nd service period (3 years long) corresponded to the presence of the FDR-AF coating. The present vessel is a bulk carrier that operates regularly between Australia and Korea. From Australia to Korea, the vessel is fully loaded to navigate in the “laden” draft, while she navigates in the “ballast” draft with the cargo hold being empty. Thus, “laden” and “ballast” voyages usually alternate, and each voyage lasts approximately half a month.

### ISO 19030 standard

In general, the propulsive performance of a ship during service is subject to many disturbances, i.e., added resistance due to rough weather conditions. It is imperative that the ship speed performance be evaluated without being affected by the additional resistance component associated with weather conditions such as wind and waves. ISO19030 has been adopted as an international standard for that purpose. The aim of this standard is to prescribe practical methods for measuring changes in ship-specific hull and propeller performance and to define a set of relevant performance indicators for hull and propeller maintenance, repair and retrofit activities^12^.

The hull and propeller performance is closely linked to the concepts of ship propulsion efficiency and ship resistance. The performance model is based on the relation between the delivered power and the total resistance, where the delivered power $P_{D}$ can be expressed as

1 $$ P_{D} = \frac{{R_{T} V}}{{\eta_{D} }},R_{T} = R_{SW} + R_{AA} + R_{AW} + R_{AH} $$

2 $$ R_{AH} = \frac{{P_{D} \eta_{D} }}{V} - \left( {R_{SW} + R_{AA} + R_{AW} } \right) $$

Here, $R_{T}$ is the total in-service resistance, $V$ is the ship speed through water, and $\eta_{D}$ is the quasipropulsive efficiency. $R_{T}$ is given as the sum of various resistance components, which are calm water resistance $R_{SW}$, added resistance due to wind $R_{AA}$, added resistance due to wave $R_{AW}$ and added resistance due to changes in hull condition (fouling, mechanical damages, bulging, coating film blistering, coating detachment, etc.) $R_{AH}$. ISO19030 aims at the quantification of $R_{AH}$ and its related speed drop during vessel service.

**Figure 4:**
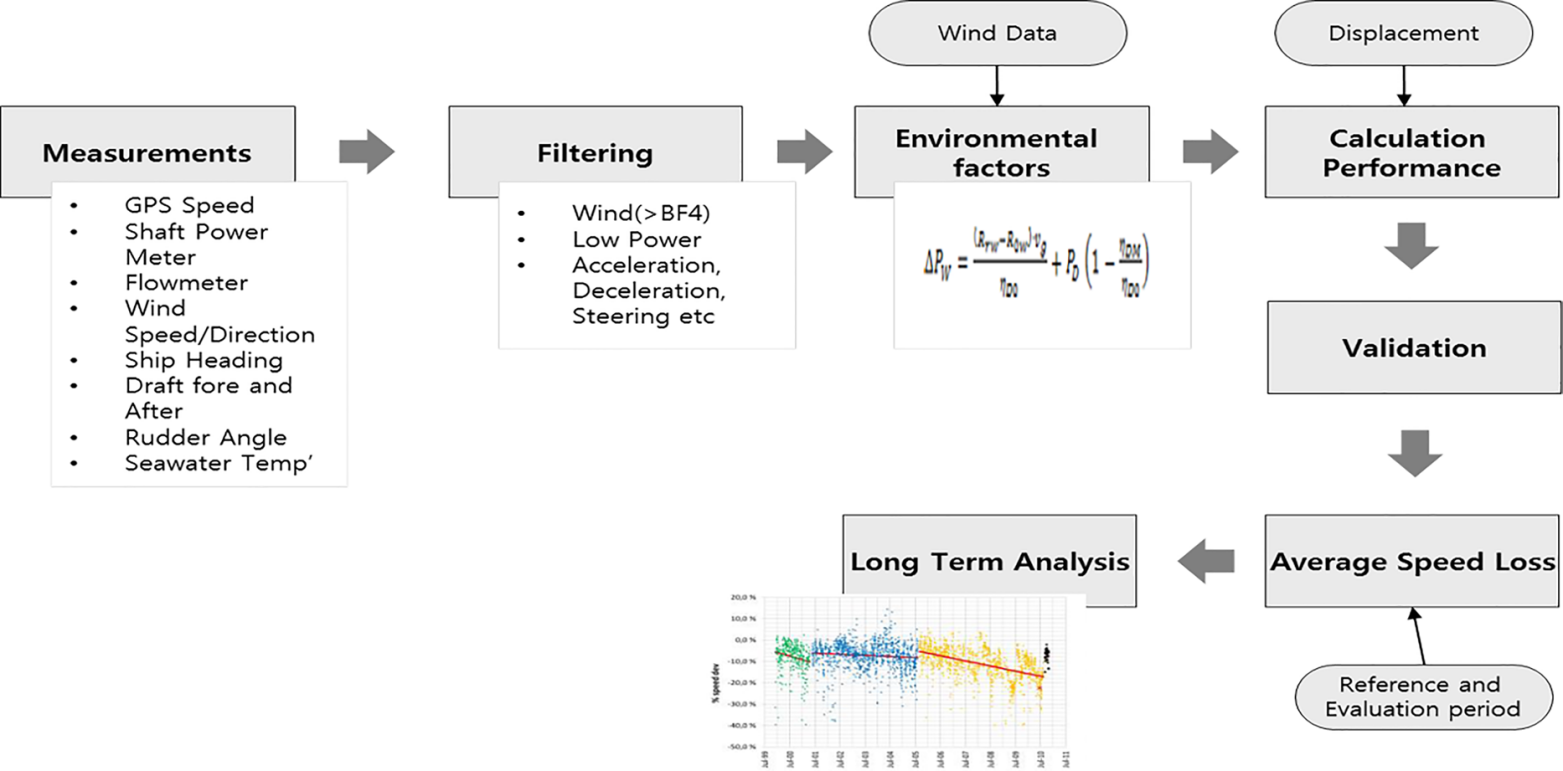
Measurement and analysis process of ISO19030.

Figure 4 illustrates the process of ISO19030, consisting of navigation data compilation, filtering, validation and analysis. The measured variables are the ground speed, ship heading, shaft power, wind speed, wind direction, drafts, seawater temperature, rudder angle, etc. These variables are supposed to be measured at a constant rate, typically every 10 s. It is well known that ship speed and shaft power tend to be affected by such various environmental variables, and influences from those variables need to be adequately removed by either filtering or compensation. The first step is the filtering. In the retrieved data set, outliers and missing values are markedly apparent, corresponding to invalid data. To this end, in consecutive, nonoverlapping blocks spanning 10 min, data for every parameter are filtered according to Chauvenet’s criterion. Table 3 lists the validation criteria for each environmental parameter. Outliers associated with environmental parameters are mostly filtered out, with the exception of wind resistance, which is compensated after the ISO15016 sea trial analysis method.

**Table 3:** Validation and filtering criteria for environmental parameters.

Parameter	Unit	Filtering	Validation	Correction
Speed over ground	[knots]	10.0 ~ 16.5	Std. dev. < 0.5 knots	
Shaft power	[kW]	4000 ~ 20,000	–	
Relative wind speed and direction	[knots], [°]	< 15.6 knots	–	ISO15016
Ship heading	[°]	–	–	
Shaft revolutions	[min^−1^]	–	Std. dev. < 3 RPM	
Static draft fore and aft	[m]	–	–	
Water depth	[m]	–	–	
Rudder angle	[°]	± 5°	Std. dev. < 1°	
Seawater temperature	[°]	over 2 °C	–	

When the valid data set and reference curve are prepared, data correction is performed. Since ISO19030 is based on correction for data that can actually be measured, the external environmental correction is applied only to wind. Correction according to wind resistance requires calculation of actual wind speed and direction based on wind speed and direction of measurement data. It is calculated according to Annex E (calculation of true wind speed and direction). To calculate the wind resistance, the wind speed and direction at the reference height of the wind tunnel test should be used.

After the effect of environmental factors such as wind and water temperature is either removed or corrected, the performance value is quantified as the percentage speed drop at the measured shaft power as follows:

3 $$ PV = \frac{{V_{m} - V_{e} }}{{V_{e} }} \times 100\left( \% \right) $$

Here, $V_{m}$ and $V_{e}$ correspond to the measured speed and the expected speed in the calm sea environment, respectively. $V_{e}$ is obtained by lookup from the speed-power curve obtained in either the speed trial or the model test. Measurements of ship-specific changes in hull and propeller performance can be used in a number of relevant performance indicators (PIs) to determine the effectiveness of hull and propeller maintenance, repair and retrofit activities. Table 4 outlines four basic hull and propeller PIs.

**Table 4:** Basic hull and propeller performance indicators (PIs).

PI	Definition
**PI-1** Dry-docking performance	Change in hull and propeller performance following the present out-docking (evaluation period) as compared with the average from previous out-dockings (reference periods)
**PI-2** In-service performance	Average change in hull and propeller performance from a period following the out-docking (reference period) to the end of the dry-docking interval (evaluation period)
**PI-3** Maintenance trigger	Change in hull and propeller performance from the start of the dry-docking interval (reference period) to a moving average at a given point in time (evaluation period)
**PI-4** Maintenance effect	Change in hull and propeller performance from before (reference period) to after a maintenance event (evaluation period)

**Figure 5:**
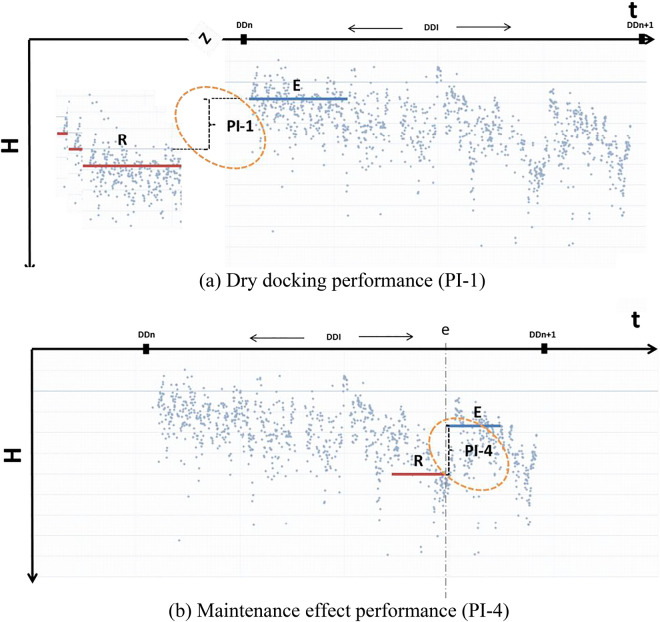
Definitions of performance indicators (PIs) considered in this study (taken from ISO 19030-1^12^).

Of the four PIs in Table 4, PI-1 and PI-4 are considered to be relevant to assess the change in speed performance of the vessel due to the change in surface condition, i.e., the AF coating and the cleaning effect after dry docking. PI-1 compares two freshly coated surface conditions with different coatings, so it is ideal for the evaluation of the effect of FDR-AF on the speed performance. On the other hand, PI-4 can provide an overall estimation of the coating effect combined with the cleaning effect. This point is addressed in greater detail in the next section. Figure 5 shows how the PIs under consideration are calculated from the time history of performance values (PVs). Here, DDn and DDn + 1 refer to the present and next dry-docking instances, respectively. R and E signify the reference period and the evaluation period, respectively.

## ISO19030 analysis results

### Temporal variation in PVs

**Figure 6:**
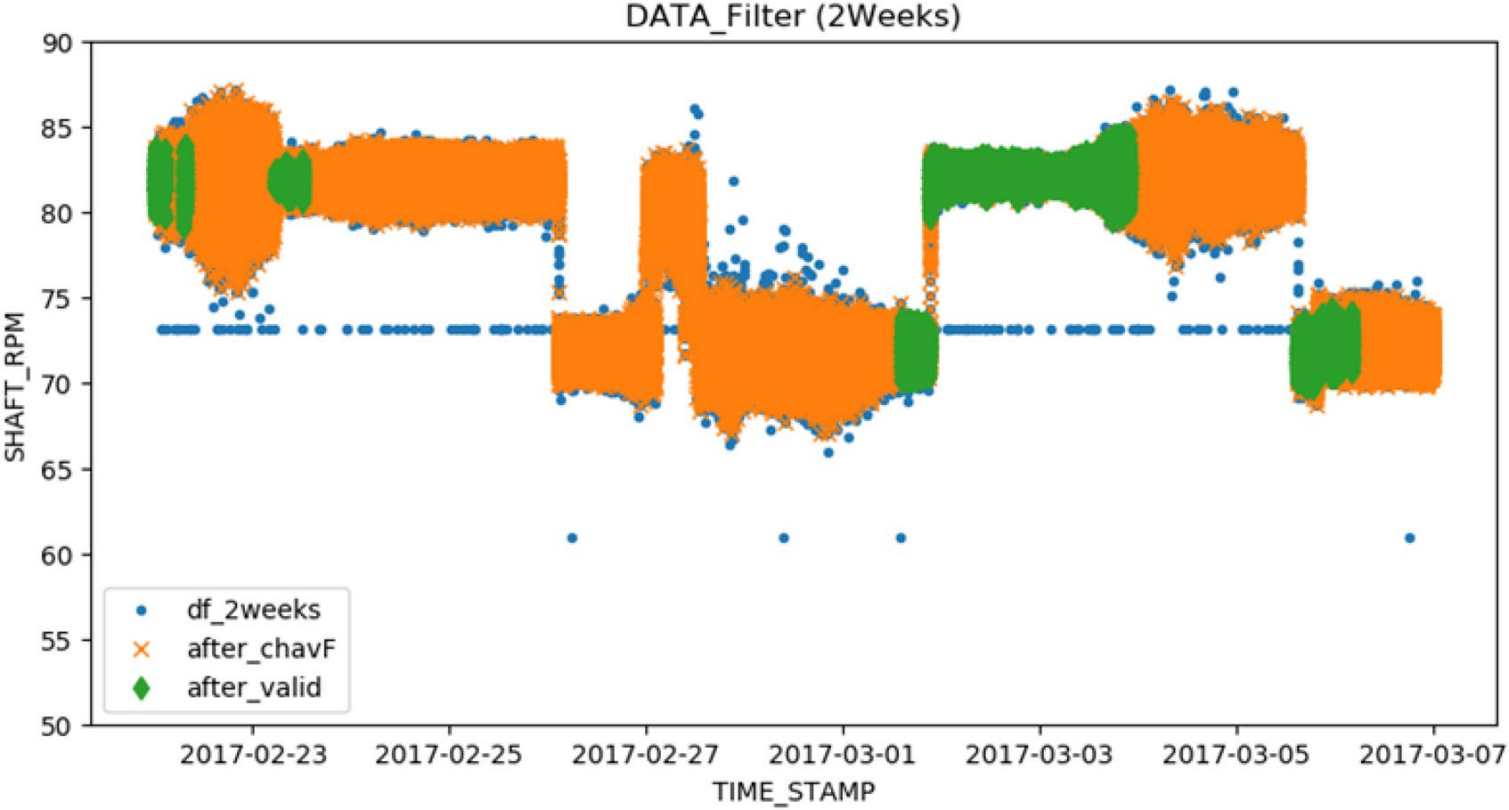
Sample plot of the measured data and validated data for 2 weeks.

All of the data were automatically acquired by the SPMS and stored in raw data form with a UTC time stamp (YYYY-MM-DDT HH: MM: SS). Operational data collected from each voyage are separated into valid and invalid data sets by the ISO19030 Chauvenet filter and validation, and the statistics of valid data sets are shown in Table 5. The numbers of instantaneous data points collected over the 5-year investigation period amounted to 2.8 millions and 2.9 millions for the laden and ballast conditions, respectively. After the filtering and validation steps, approximately 52% and 66% of the original data respectively remained to form valid data sets for each load condition. Considering that there are 46 voyages for each load condition, this implies that the final valid data set for each voyage consists of at least 33,000 instantaneous data points. Example results of data outlier filtering and the validation process for one voyage of data are depicted in Fig. 6. Blue circles are the original data, orange x marks are the data set that satisfied Chauvenet’s criterion, and green diamonds are the final valid data set.

**Table 5:** Percentage of valid operational data after filtering and validation.

Operating condition	Laden condition	Ballast condition
Original data	100% (2.9 M)	100% (2.8 M)
**Filtering**		
Chauvenet’s criterion	10.8%	10.4%
Validation	37.4%	23.3%
Valid data	51.8%	66.3%

**Figure 7:**
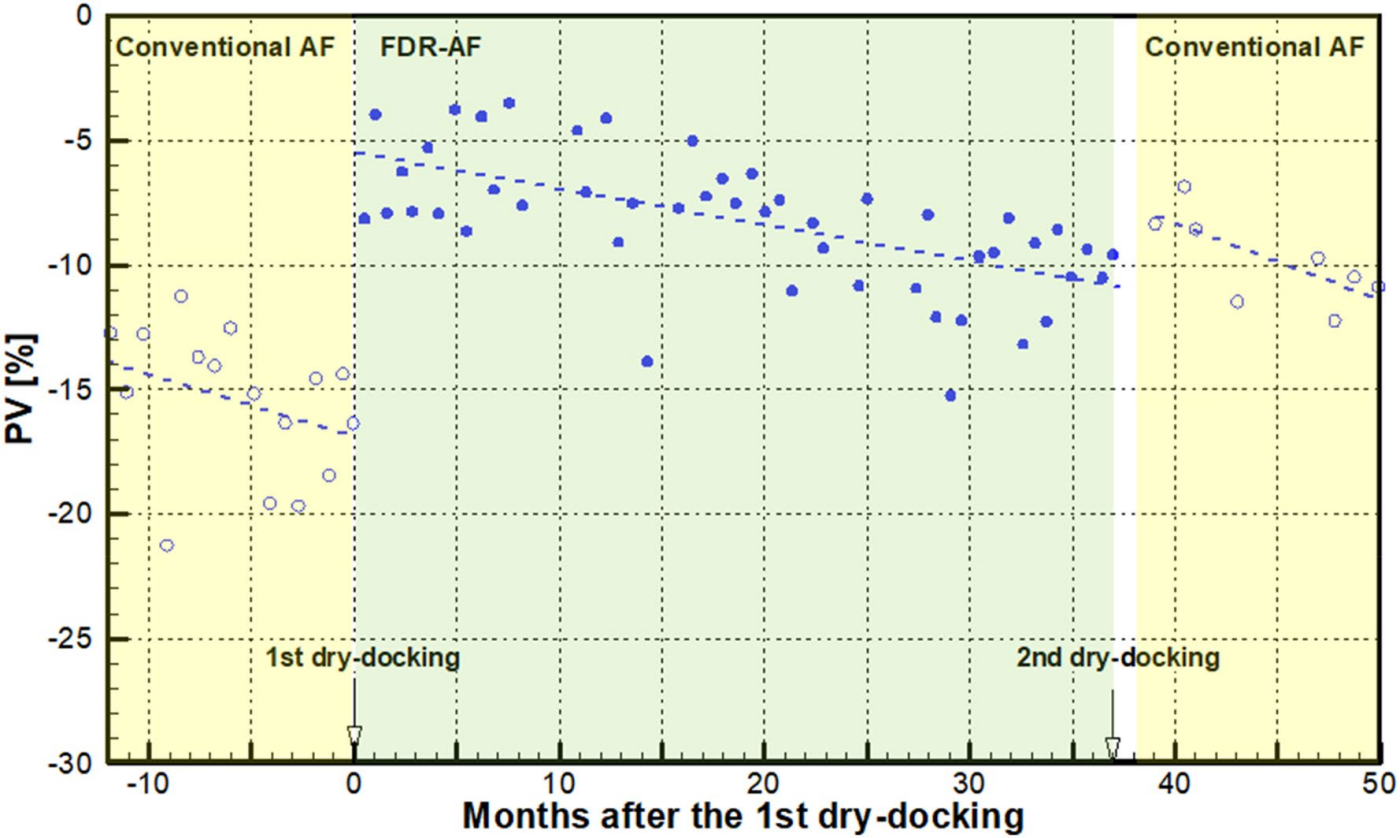
Temporal variation in the voyage-averaged PVs.

Every valid dataset contains instantaneous information regarding speed, shaft power, draft and weather. After the power is corrected for the wind resistance and draft change, it can be compared with the speed-power curve in the calm sea environment to give the expected speed $V_{e}$. Using Eq. (3), this value is subsequently used to calculate the instantaneous PV. Thus, millions of instantaneous PVs are obtained, which makes it impractical to plot the PVs as a function of time. Consequently, the average was taken of the instantaneous PVs during each voyage. This is reasonable in that during each voyage, the operating conditions of the vessel do not change considerably, thereby giving rise to a coherent data set. Figure 7 shows the temporal variation in such voyage-averaged PVs over the entire 5-year analysis period.

The horizontal axis of Fig. 7 shows the time in terms of months after the 1st dry docking. Therefore, the negative values of months correspond to the 1st service period before then. The 1st dry docking is marked at month 0, and the 2nd is marked at month 37. They separate three service periods with varying AF coatings: 1st with conventional AF, 2nd with FDR-AF and 3rd with conventional AF. Each symbol in Fig. 7 represents the abovementioned voyage-averaged PV value. This negative PV value corresponds to the percentage speed drop during operation. A brief perusal of Fig. 7 clearly indicates that the present vessel exhibits a significant level of speed drop, ranging from 5 to 20%, throughout the entire analysis period. Despite the scatter of PV values, the PV clearly shows a gradual decrease over time, which corresponds to the temporal degradation of the speed performance of the vessel.

In Fig. 7, three service periods are noted with different shades associated with the AF coatings involved. Regarding the 2nd service period (months 0 through 37), a closer comparison between the initial and final values of the dash dot trend line reveals that the speed dropped by approximately 5% from the initial value − 6% at month 0 to the final value − 11% at month 37. The underlying mechanism is the deterioration of the surface condition, which is discernible by comparing photographs taken after the 1st dry docking in Nov. 2015 (Fig. 2) and those before the 2nd dry docking in Dec. 2018 (Fig. 3). After 3 years of service, the AF coating is sporadically peeled off or damaged. The peeling-off of the coating can be attributed to the natural erosion process of the SPC resin. The coating damage can be caused by berthing to the pier. Although the tendency of coating deterioration is evident from the temporal decrease in the PVs, its quantification by the above end-to-end comparison of the PVs could be misleading because individual voyage performance might be subject to unknown disturbances, such as seasonal variations in the environment. The PI, which is derived from long-term averages of PVs, can give an unbiased estimate of coating performance. The PI is addressed in greater detail in the next section.

Another observable feature in Fig. 7 is the maintenance effect, i.e., the abrupt increase in the PVs after dry docking. Observation of Figs. 2 and 3 confirms that dry docking returned the deteriorated hull surface condition back to the pristine condition. Consequently, the speed performance quantified by PV should naturally improve, which is termed the maintenance effect. Two dry dockings occurred, and the associated PV jumps were as follows: for the 1st dry docking, PV increased by more than 10%P, while the increase in the PVs coincident with the 2nd dry docking was approximately 3%P.

During dry docking, the deteriorated AF coating during the previous operation is removed, and a new AF coating is applied to the entire underwater area. Therefore, there must be a hull cleaning effect to improve speed performance. The abovementioned differences in the increase in PV, however, suggest that there is an additional effect associated with the coating. At the 1st dry docking, the conventional AF coating was replaced by the low-friction FDR-AF. At the 2nd dry docking, an AF coating was restored. If there exists a speed improvement effect of FDR-AF that increases the PV value, then it is expected to act in the opposite direction at each dry docking. At the 1st dry docking, this will act in conjunction with the hull cleaning effect to make the maintenance effect greater, while it will counteract the hull cleaning effect at the 2nd dry docking. The significant difference between PV changes strongly supports that this is the case with the present vessel. In the next section, the coating effect as well as the hull cleaning effect is quantified in connection with the PI.

### PIs

In the previous section, the speed performance degradation by coating deterioration, the hull cleaning effect and the coating effect were noted by an end-to-end point comparison of the PVs. Individual PV values, which are the average taken during a single voyage, are subject to environmental disturbances such as currents and waves. This is because the effects from those disturbances are not compensated in ISO19030. The average taken for a longer period (e.g., one year) becomes essentially uncorrelated with such voyage-specific disturbances and seasonal variations, thereby enabling the quantitative assessment of vessel speed performance. This is the underlying idea of the PI. In this study, the reference period and the evaluation period are taken as long as one year.

**Figure 8:**
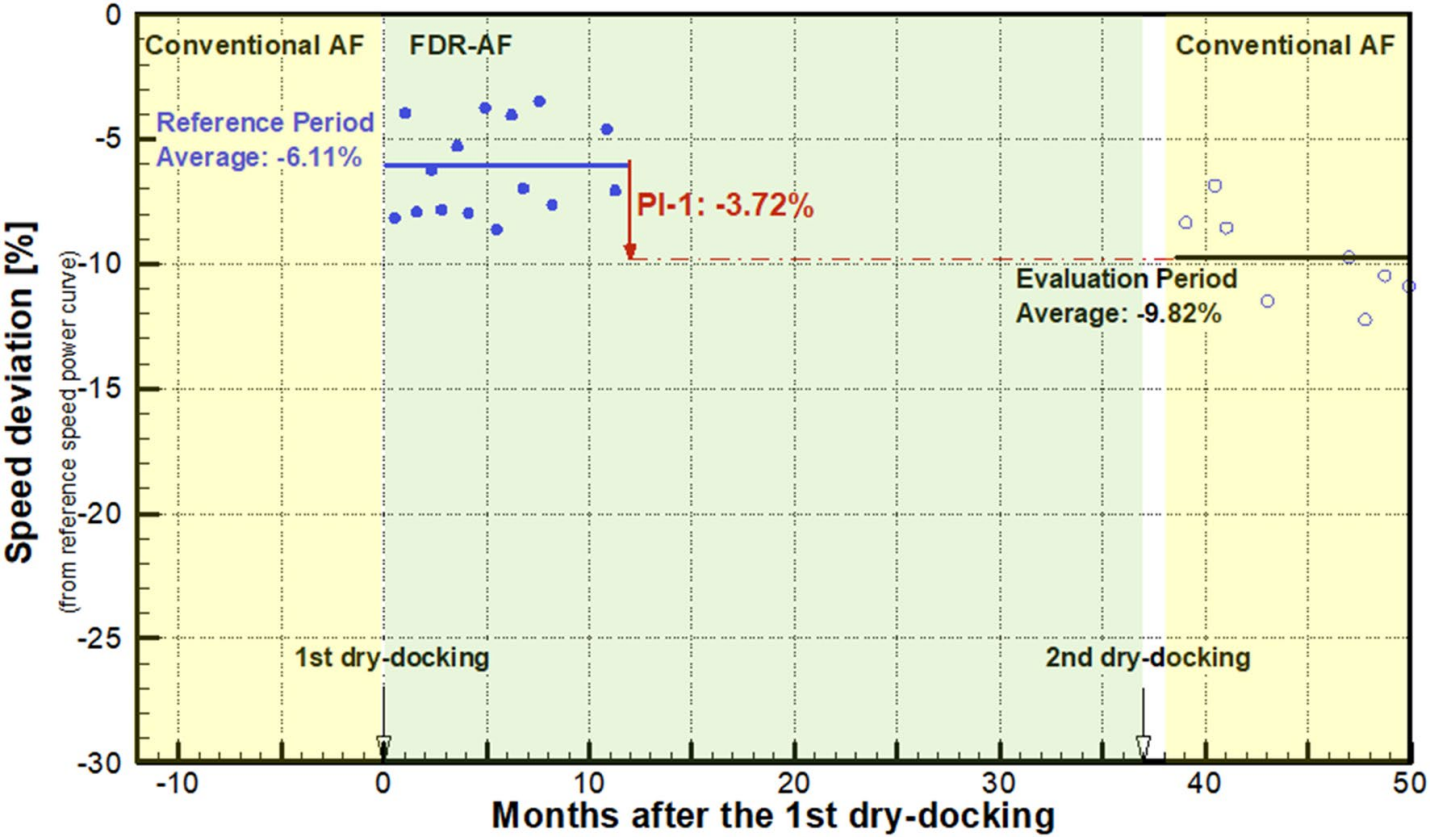
The AF coating effect quantified by the dry-docking performance (PI-1).

Figure 8 depicts the dry docking performance (PI-1) for the 2nd dry docking. Here, the evaluation period is the first year (months 38 through 50) of the 3rd service period with the conventional AF coating. On the other hand, the reference period is the first year (months 0 through 12) of the 2nd service period with the FDR-AF coating. It is notable that those two periods are in the same, freshly coated condition except that different coatings are applied. Consequently, this is an ideal situation to quantify the effect of a coating, if any, on the speed performance of the vessel. The PI-1 of − 3.72% in Fig. 8 indicates that the present FDR-AF coating led to a speed improvement of 3.72% compared to the conventional AF coating. This is termed the coating effect of the present FDR-AF coating.

**Figure 9:**
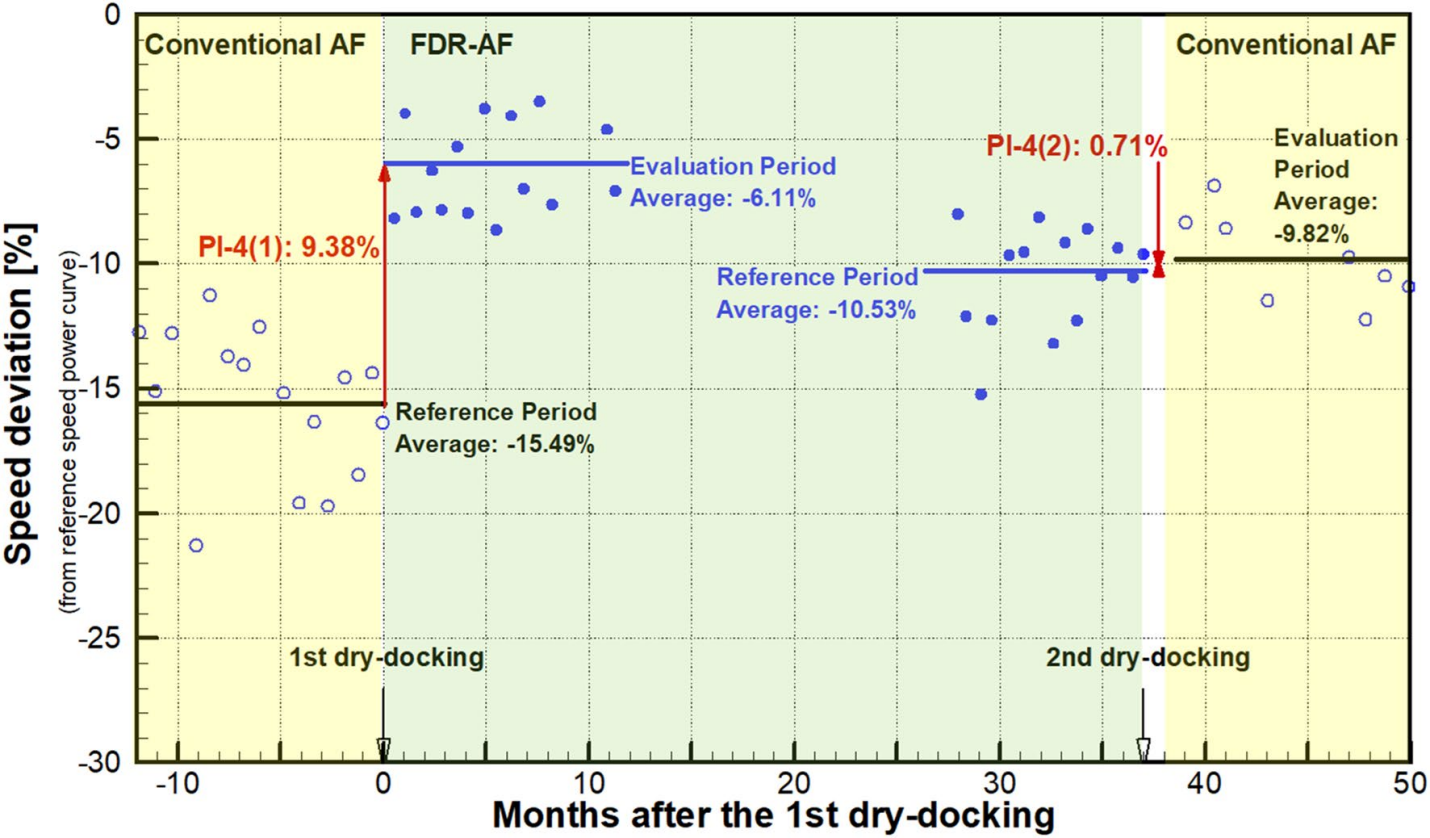
The maintenance effect (PI-4).

The maintenance effect (PI-4) calculated for the two dry dockings is plotted in Fig. 9. PI-4(1) associated with the 1st dry docking is calculated as 9.38%, while PI-4(2) for the 2nd dry docking is only 0.71%. Similar behaviors are also observed from the comparison of PV in the previous section, which implies that the counteracting relationship between the coating effect and the hull cleaning effect is also confirmed on a long-term basis. Consequently, the small value of PI-4(2) for the 2nd dry docking can be explained as follows: the performance improvement expected from a fresh coating is offset by the performance degradation by adopting the conventional AF, which is now found to be relatively inferior to the previously applied FDR-AF by 3.72%.

The coating effect, which is defined as the relative speed improvement by the present FDR-AF over the conventional AF, has been quantified as 3.72% from PI-1 analysis. It is reasonable to assume that the same amount of coating effect should act pertaining to the maintenance effect (PI-4). As mentioned earlier, replacement with a fresh AF coating during dry docking leads to an improvement in speed performance, which is called the hull cleaning effect. For the 1st dry docking in which the conventional AF was replaced by FDR-AF, the coating effect adds up to the hull cleaning effect to make PI-4(1). On the other hand, the coating effect should be subtracted from the hull cleaning effect for the 2nd dry docking pertaining to PI-4(2).

A closer inspection of Figs. 2 and 3 indicates that the surface condition before the 1st dry docking in Fig. 2 is more damaged than that for the 2nd dry docking in Fig. 3. Since the hull cleaning effect stems from the differences in surface conditions before and after dry docking, differences in initial conditions affect the hull cleaning effect. To compensate for the effect of varying degrees of initial coating damage before dry docking, an additional term needs to be introduced to take into account the damage maintenance effect. This is termed the “damage recovery effect”.

Summarizing these terms, the maintenance effect (PI-4) for each dry docking can be modeled as follows:

4 $$ {\text{PI}} - 4\left( 2 \right) = - {\text{ Coating Effect}} + {\text{ Hull Cleaning Effect}} $$

5 $$ {\text{PI}} - 4\left( 1 \right) = {\text{ Coating Effect}} + {\text{ Hull Cleaning Effect}} + {\text{Damage Recovery Effect}} $$

**Figure 10:**
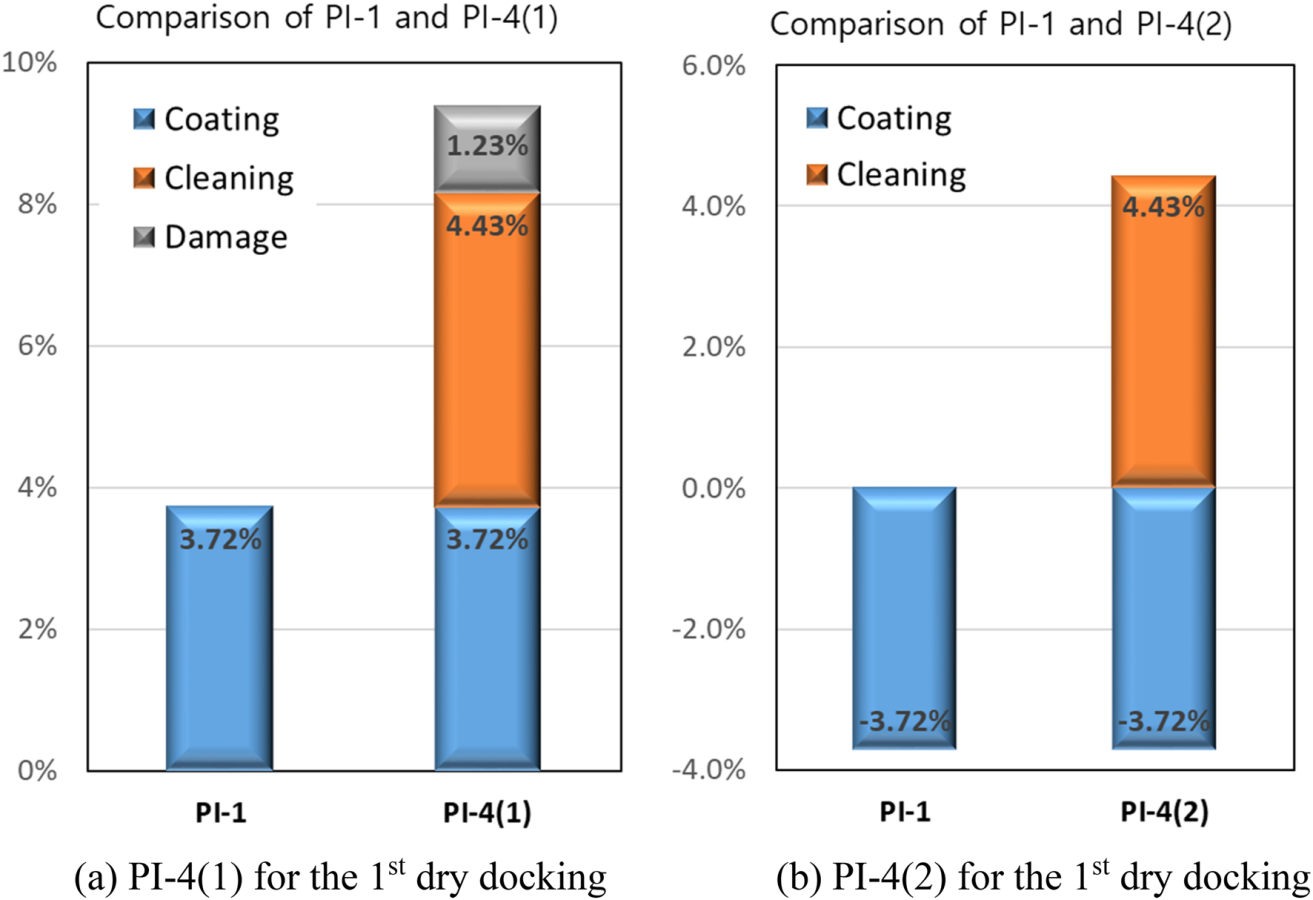
Analysis of contributing effects to the maintenance effect (PI-4) for each dry docking.

For the 2nd dry docking, the damage recovery effect is assumed to be absent in the maintenance effect PI-4(2). This is based on the observation results before the 2nd dry docking in Fig. 3. As given in Fig. 9, PI-4(1) = 9.38%, and PI-4(2) = 0.71%. Additionally, the coating effect is found to be 3.72%. Solving Eqs. (4) and (5) with these values gives a hull cleaning effect of 4.43% and a damage recovery effect of 1.23% for the present vessel. Figure 10 illustrates how the abovementioned effects are combined to give performance indicators pertaining to each dry docking.

It is worthwhile to mention the results of a speed performance analysis by the authors for a sister vessel^13^. This vessel has the same dimensions as the present vessel and was operated in a similar manner. The most distinctive difference of the sister ship is that the same conventional AF coating was adopted for two consecutive service periods. PI-1 for this ship was found to be only − 0.18%, which is consistent with the expectation that the coating effect should vanish for identical coatings. In this case, PI-4 should contain only the hull cleaning effect, which was observed to be 3.63%. This value is comparable to the 4.43% obtained for the present vessel. In summary, the results obtained from the sister vessel support the present model to account for the variance in the maintenance effect.

Finally, it would be informative to correlate the speed improvement effect of the present FDR-AF coating with the possible power (fuel) saving effect. Townsin and Kwon^14^ proposed a correlation between the percentage speed drop ${\Delta V}/V$ and the power increase due to added resistance ${\Delta }P/P$ as follows:

6 $$ \frac{{{\Delta }P }}{P} = \left( {n + 1} \right)\frac{{{\Delta }V }}{V} $$

Here, $n$ is determined by the vessel type and draft; $n = 1.91$ for the laden draft for a very large crude oil carrier (VLCC) and $n = 2.40$ for the VLCC ballast draft. Considering that the present vessel operates half in laden and half in ballast draft, the average value $n = 2.16$ seems to be reasonable. This means that the power saving percentage due to the present FDR-AF coating would correspond to approximately 3.16 times the speed increase percentage in the present vessel. Inserting the coating effect 3.72% into Eq. (6) gives $\frac{{{\Delta }P }}{P} = 11.7\%$, which characterizes the power (fuel) saving effect of the present FDR-AF coating. It is notable that this value is in reasonable agreement with the 18.2% power saving effect predicted by Lee and Park^15^, who extrapolated the skin friction laboratory measurement results of the present FDR-AF to full-scale vessel resistance based on the similarity transform method of Granville^16^ and Schultz^17^.

The combined hull cleaning effect and the damage recovery effect involved in PI-4(1) caused the ship speed to drop by 5.66%. Application of the correlation leads to a power increase of 17.9%. This particular value is similar to the 22.54% increase in the total resistance coefficient $\left( {\% \Delta C_{T} } \right)$ for a 290 m-long bulk carrier subject to “orange peel” roughness, which was predicted by Hakim et al*.*^18^.

## Conclusions

A novel FDR-AF coating, which was based on the polymer drag reduction principle, was developed and found to be promising in terms of AF efficiency as well as long-term drag-reducing capability. The coating was then commercially manufactured and applied to the entire underwater surface of a 176 k DWT bulk carrier during redocking in December 2015. Since November 2014, the propulsion performance and weather conditions have been recorded over five years, which can be subdivided into three service periods with different adopted AF coatings.

These service periods were carefully selected to enable an isolated comparison of speed performances of the present vessel with AF coatings being varied. Based on the ISO 19030 standard, the in-service performance of the 176 k DWT bulk carrier was analyzed to assess the speed improvement due to the present FDR-AF coating. The present coating was found to be accountable for a significant speed increase of 3.72% over the conventional AF coating, which is termed the coating effect. In addition, other effects, such as the hull cleaning effect and damage recovery effect, amount to speed improvements of 4.43% and 1.23%, respectively. The speed improvement by the present FDR-AF coating is equivalent to a power (fuel) saving effect of 11.7%.
